# Neuroprotective effect of acetoxypachydiol against oxidative stress through activation of the Keap1-Nrf2/HO-1 pathway

**DOI:** 10.1186/s12906-024-04474-6

**Published:** 2024-04-25

**Authors:** Yu Qi, Ge Liu, Shengjie Jin, Rong Jian, Ziqiang Zou, Chenjing Wang, Yuanlong Zhang, Min Zhao, Haoru Zhu, Pengcheng Yan

**Affiliations:** 1 School of Traditional Chinese Medicine, Wenzhou Medical University, Wenzhou, Zhejiang 325035 People’s Republic of China; 2 Department of pharmacy, Yongkang First People’s Hospital Affiliated to Hangzhou Medical College, Yongkang, 321300 People’s Republic of China; 3 School of Pharmaceutical Sciences, Wenzhou Medical University, Chashan University Town, Ouhai District, Wenzhou, Zhejiang 325035 People’s Republic of China

**Keywords:** Acetoxypachydiol, Neuroprotective effect, Keap1-Nrf2/HO-1 pathway, Oxidative stress

## Abstract

**Background:**

Excessive oxidative stress in the brain is an important pathological factor in neurological diseases. Acetoxypachydiol (APHD) is a lipophilic germacrane-type diterpene extracted as a major component from different species of brown algae within the genus *Dictyota*. There have been no previous reports on the pharmacological activity of APHD. The present research aims to explore the potential neuroprotective properties of APHD and its underlying mechanisms.

**Methods:**

The possible mechanism of APHD was predicted using a combination of molecular docking and network pharmacological analysis. PC12 cells were induced by H_2_O_2_ and oxygen–glucose deprivation/reoxygenation (OGD/R), respectively. Western blot, flow cytometry, immunofluorescence staining, and qRT-PCR were used to investigate the antioxidant activity of APHD. The HO-1 inhibitor ZnPP and Nrf2 gene silencing were employed to confirm the influence of APHD on the signaling cascade involving HO-1, Nrf2, and Keap1 in vitro.

**Results:**

APHD exhibited antioxidant activity in both PC12 cells subjected to H_2_O_2_ and OGD/R conditions by downregulating the release of LDH, the concentrations of MDA, and ROS, and upregulating SOD, GSH-Px, and GSH concentrations. APHD could potentially initiate the Keap1-Nrf2/HO-1 signaling cascade, according to the findings from network pharmacology evaluation and molecular docking. Furthermore, APHD was observed to increase Nrf2 and HO-1 expression at both mRNA and protein levels, while downregulating the protein concentrations of Keap1. Both Nrf2 silencing and treatment with ZnPP reversed the neuroprotective effects of APHD.

**Conclusions:**

APHD activated antioxidant enzymes and downregulated the levels of LDH, MDA, and ROS in two cell models. The neuroprotective effect is presumably reliant on upregulation of the Keap1-Nrf2/HO-1 pathway. Taken together, APHD from brown algae of the genus *Dictyota* shows potential as a candidate for novel neuroprotective agents.

**Supplementary Information:**

The online version contains supplementary material available at 10.1186/s12906-024-04474-6.

## Introduction

Oxidative stress is a damaging condition resulting from an imbalance between the generation of oxygen-derived free radicals and the body’s antioxidant capability, leading to excessive production of reactive oxygen species (ROS) [1]. Stroke, a neurological disease related to oxidative stress, has a high incidence and is among the most prevalent health conditions worldwide [2]. Ischemic stroke accounts for the majority of stroke cases. During the progression of an ischemic stroke, a significant amount of ROS is produced in brain tissue ischemia and reperfusion, exacerbating oxidative stress [3]. Consequently, the search for effective antioxidants is a crucial therapeutic strategy in treating cerebral ischemia injury.

The Keap1/Nrf2 cascade functions as an intrinsic defense system against oxidative stress for redox reactions [4, 5]. Keap1 acts as an inhibitory controller of Nrf2, typically binding to Nrf2 in cells to form a complex that inhibits Nrf2 activity, with Nrf2 forming a stable trimer with Keap1 [6, 7]. Under conditions of cellular oxidative stress, Keap1 releases Nrf2, which then steadily migrates into the nucleus [8]. Through attachment to the antioxidant response element (ARE) present in the promoter regions of genes encoding antioxidant proteins, such as HO-1 and the glutamate-cysteine ligase modifying subunit (GCLM), it can stimulate the production of antioxidant agents such as superoxide dismutase (SOD), glutathione (GSH) and glutathione peroxidase (GSH-Px). This bolsters cellular defenses against oxidative stress, suppresses the generation of lactate dehydrogenase (LDH), malondialdehyde (MDA) and reactive oxygen species (ROS), and consequently provides a safeguarding effect within the cells [9]. The Keap1-Nrf2/HO-1 signaling cascade is regarded as the “switch” of the endogenous antioxidant system and can effectively mitigate damage from cerebral ischemia [10]. It has been proven that reinforcing the interaction between Keap1 and Nrf2 can reduce cerebral infarct volume and ROS generation in vivo [11]. The Keap1-Nrf2/HO-1 cascade is also implicated in various processes, such as mitophagy and cell apoptosis, thereby offering neuroprotection against several nervous system diseases [12–14]. Although numerous natural substances have been identified that regulate the Keap1-Nrf2/HO-1 cascade and show potential in mitigating cerebral ischemic injury, there is currently a lack of such compounds approved for the market. Therefore, it is necessary to continue searching for effective Nrf2 activators from natural sources to develop neuroprotective drugs specifically for cerebral ischemia.

The ocean is a treasure trove with potential drug utilization value, yet the exploitation of marine organisms is still limited. Consequently, the development of new drugs from marine resources has become a focal point of scientific research. Currently, there are approximately 17 marine-derived drugs approved by the FDA for international marketing [15], primarily used in anti-tumor and anti-viral treatments [16, 17]. However, research on the neuroprotective properties of marine natural products is scarce. Studies have demonstrated that certain algal extracts exhibit promising neuroprotective activities [18], making them valuable for further exploration in the development of neuroprotectants. In recent years, our research has continued to explore the neuroprotective active ingredients in marine brown algae, identifying several diterpenes with antioxidant and neuroprotective effects [19, 20]. The genus *Dictyota* (family Dictyotaceae) is an important source of medicinal algae, documented in the Chinese Marine Materia Medica for its use in detoxification and inflammation suppression. It has been reported that some secondary metabolites of the genus *Dictyota* possess a multiplicative neuroprotective effect [19, 21, 22]. Acetoxypachydiol (APHD), a lipophilic germacrane-type diterpene, has been derived from the organic extracts of *Dictyota* algae as a characteristic component with high content [23, 24]. However, the neuroprotective effect of APHD has not yet been reported. This study aims to explore the neuroprotective properties of APHD in treating cerebral ischemic damage and its underlying mechanisms.

In the current study, a series of comprehensive *in silico* analyses, including molecular docking and network pharmacology, were employed to elucidate the biological mechanism of APHD, followed by in vitro confirmation of its antioxidant activity. We established an H_2_O_2_-induced PC12 cell model and a PC12 cell model subjected to oxygen–glucose deprivation/reoxygenation (OGD/R) to investigate the neuroprotective effect and corroborate the *in silico* predicted mechanisms of APHD from different perspectives. This study demonstrated that APHD exerts an antioxidant effect and protects the brain from ischemic damage, potentially through the activation of the Keap1-Nrf2/HO-1 signaling cascade.

## Materials and methods

### Materials

APHD with a purity of over 98.5% was extracted and isolated from *Dictyota coriacea*, which was collected off the coast of Nanji Island, Wenzhou, Zhejiang Province, China, in May 2018. The isolation and extraction methods are as reported in previous literature [24]. Tert-butylhydroquinone (TBHQ) was supplied by MedChemExpress (USA). Antibodies targeting Keap1 and HO-1 were obtained from Abcam (MA, USA). Antibodies targeting Nrf2 and β-actin were procured from Proteintech Group (Wuhan, China).

### The APHD related targets identification

SwissTargetPrediction (http://www.swisstargetprediction.ch/, accessed on September 30, 2022) and SuperPred (https://prediction.charite.de/, accessed on September 30, 2022), two open-source webservers, were used to identify potential targets affected by APHD. After integrating and de-duplicating the predicted targets, their gene names were standardized and verified on the Universal Protein Resource (UniProt, https://www.uniprot.org/, accessed on October 1, 2022).

### Protein-protein interaction (PPI) network

The STRING database (http://string-db.org, accessed on October 1, 2022) was used to construct the PPI network (confidence score > 0.9) for the APHD-related targets. This aimed to explore the interactions among the targets. Hub targets were identified within the PPI network based on topological features such as degree, neighborhood connectivity (NC), betweenness centrality (BC), and closeness centrality (CC), calculated using the CytoNCA plugin in Cytoscape (version 3.10.1). Only proteins with degree, NC, BC, and CC values at or above their respective medians were considered hub targets. All networks were visualized using Cytoscape.

### Enrichment analysis

DAVID (http://david.ncifcrf.gov, accessed on October 9, 2022) was used for the enrichment and annotation of APHD-related target genes using KEGG, GO, and WikiPathways methods. In the GO analysis, target genes were categorized within MF, CC, and BP. The top 10 terms from the GO analysis for MF, CC, and BP were visualized in a single bubble chart using R software (version 4.3.1). Similarly, the top 20 terms from KEGG and WikiPathways analyses were represented in bubble charts using R software.

### Molecular docking

We utilized the PDB (http://www.rcsb.org, accessed on October 12, 2022) to determine the 3D protein structures of hub targets. PubChem (https://pubchem.ncbi.nlm.nih.gov, accessed on September 30, 2022) provided the 3D structure of APHD. AutoDock Vina, an open-source software, was used to optimize and analyze the docking conformations of ligand-macromolecules. Docking affinities were evaluated based on binding energy (-kcal/mol), and the best docking results were visualized using PYMOL (open source, version 2.4.0a0).

### Cell culture

PC12 cells were obtained from the Cell Resource Center of the Shanghai Academy of Biological Sciences, which is affiliated with the Chinese Academy of Sciences. These cells were cultured in a mixture of Dulbecco’s Modified Eagle’s Medium (DMEM, Gibco, USA), 1% penicillin-streptomycin (Gibco, USA), and 10% FBS (Gibco, USA). The cell culture was maintained at 37 °C in an incubator with a 5% CO_2_ atmosphere.

The H_2_O_2_-induced PC12 cell model: PC12 cells were pretreated with various concentrations of APHD or 10 µM TBHQ for 6 h, followed by exposure to 500 µM H_2_O_2_ for an additional 24 h.

The PC12 cell model subjected to OGD/R: PC12 cells were seeded in a 96-well plate at a density of 1 × 10^4^ cells per well. After adhesion, the cells were pretreated with different concentrations of APHD or 10 µM TBHQ for 6 h. Then, the supernatant was removed, and D-glucose-free DMEM was added before placing the cells in a hypoxic chamber for 5 h. Subsequently, the medium was replaced with a complete medium containing the drugs, and the culture was continued for 24 h under normal conditions.

### MTT assay

After incubation, the medium was discarded, and 100 µL of MTT (Solarbio, Beijing, China) solution (5 mg/mL) was added to each well, followed by incubation for 4 h. The 96-well plate was centrifuged at 1000 rpm for 10 min, the supernatant was carefully removed, and 150 µL of DMSO was added to each well. The formazan dye was dissolved by shaking for 2 min, and the absorbance (OD) at 490 nm was measured with a microplate reader. The cell survival rate was calculated using the formula: Cell Survival Rate (%) = (OD of experimental group / mean OD of control group) × 100%.

### Detecting of intracellular ROS, LDH release, MDA, GSH, GSH-px, and SOD activities

The concentration of intracellular ROS was detected by flow cytometry using 2,7-Dichlorofluorescin Diacetate (DCFH-DA) [19]. After drug treatment, 1 mL serum-free DMEM containing DCFH-DA fluorescent probe was added to the cells and incubated in the dark for 30 min. The culture medium was removed by suction and PBS was added to make a cell suspension. After washing and centrifugation, cells were re-suspended using PBS, and relative fluorescence intensity was measured by flow cytometry.

PC12 cells were seeded in 6-well plates at a number of 4 × 10^5^ cells per well. After drug treatments, the supernatant and cell lysate were collected. The release of LDH and the activities of MDA, GSH, GSH-px, and SOD were measured using assay kits (Nanjing jiancheng Bioengineering Institute, Nanjing, China) according to the manufacturer’s instructions.

### qRT-PCR

The incubated cells were washed with PBS, and mRNA was extracted using a Trizol: chloroform (5:1) mixture. The reverse transcription system was prepared on ice according to the HiScript III All-in-one RT SuperMix Perfect for qRT-PCR kit (TAKARA, Japan) instructions. PCR reaction mixtures were prepared on ice using the TB Green Premix Ex Taq II kit (TAKARA, Japan), mixed, centrifuged, and subjected to a fluorescence quantitative PCR reaction in a PCR instrument. The results were analyzed using the 2^−ΔΔCt^ method. The primers were as follows: for *Nrf2*, 5’-CTGTGATCTGTCCCTGTGTAAA-3’ (Forward) and 5’-CTGTGATCTGTCCCTGTGTAAA-3’ (Reverse); for *HO-1*, 5’-GATGGCCTCCTTGTACCATATC-3’ (Forward) and 5’- AGCTCCTCAGGGAAGTAGAG − 3’ (Reverse); for *β-actin*, 5’-ACAGGATGCAGAAGGAGATTAC-3’ (Forward) and 5’-ACAGTGAGGCCAGGATAGA-3’ (Reverse).

### Western blot

Western-blot assay was established referred to previously reported protocols [20]. Briefly, PC12 Cells were seeded in 6-well plates at a density of 4 × 10^5^ cells per well. After treatment with drugs, cell lysate consisting of RIPA and PMSF was added to each well. Protein concentrations were measured using a BCA protein assay kit. Proteins were separated by SDS-polyacrylamide gel electrophoresis and transferred to PVDF membranes. The membranes were blocked using 5% non-fat milk powder and incubated with primary and secondary antibodies sequentially. The membranes were then scanned using a gel imaging system.

### Immunofluorescence

Cells were seeded on cell slides and placed in 24-well plates at a number of 1 × 10^5^ cells per well and then processed as described [20]. After treatments with drugs, the cells were fixed with paraformaldehyde, blocked with goat serum, incubated with an anti-Nrf2 antibody overnight, and then with a secondary antibody for 1 h in the dark. Finally, DAPI and fluorescent mounting media were added, and the cells were observed under a microscope.

### Cell transfection

PC12 cells were seeded in a 96-well plate at a density of 1 × 10^4^ cells and then processed as described [25]. Lipo2000 (Thermo Fisher, USA) and Nrf2-siRNA (GeneCopoeia, Shanghai China) were gently mixed and incubated at room temperature for 20 min to form a complex. The mixture was then added to the incubated cells, which were cultured for 6 h. Subsequently, the culture medium was replaced with complete medium, and incubation continued for another18 hours.

### Statistical analysis

Data were subjected to statistical evaluation using SPSS Statistics Version 20.0 and are presented as mean ± standard deviation (SD). Intergroup comparisons were made using one-way analysis of variance (ANOVA), and Levene’s Test was utilized for homogeneity of variances. If the p-value of homogeneity ≥ 0.05, SNK analysis was performed to compare inter-group variables. If the p-value of homogeneity < 0.05, Dunnett’s T3 test was compared between every two groups.

## Results

### APHD relieves the oxidative stress injury in H_2_O_2_-induced PC12 cells.

**Fig. 1:**
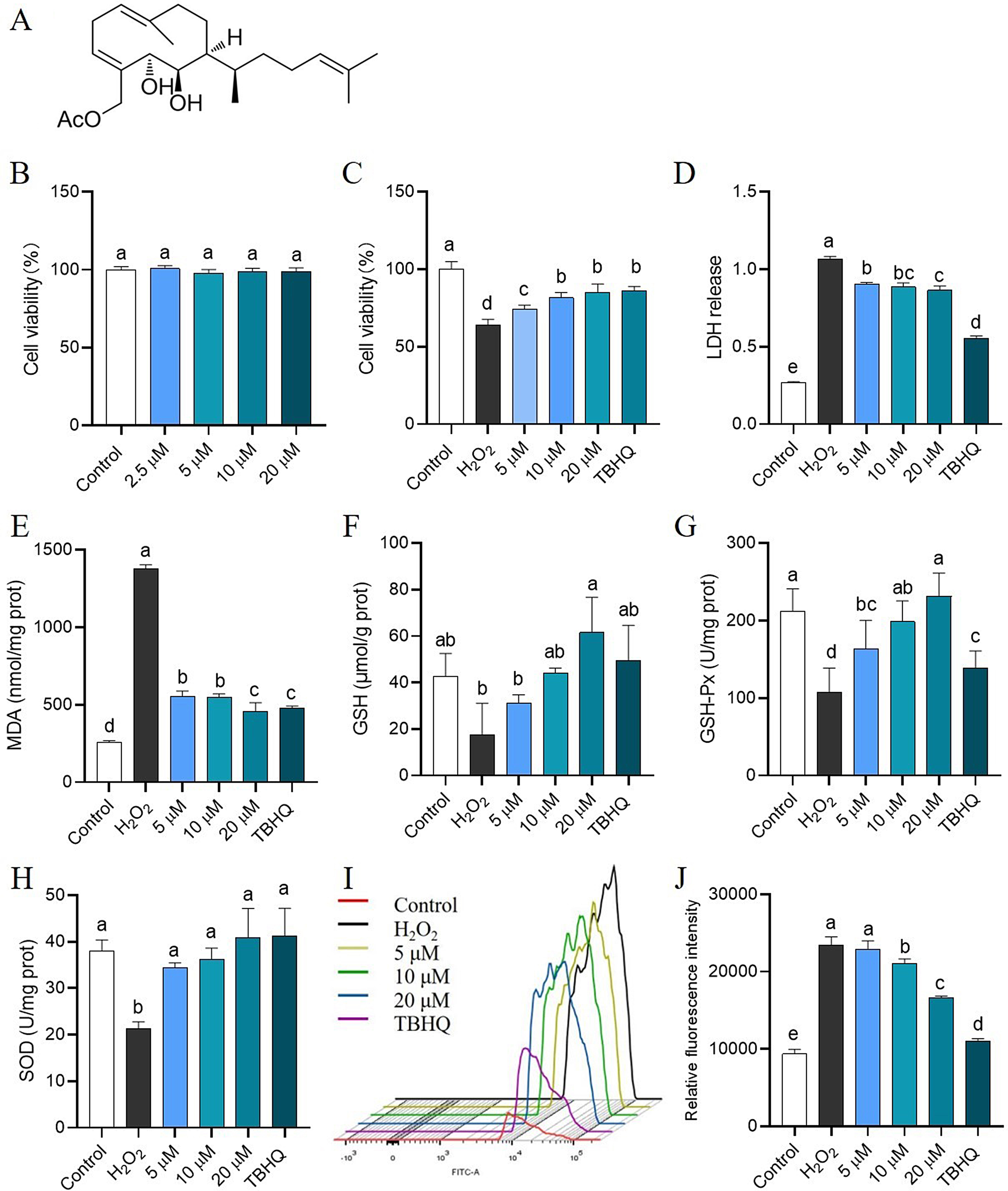
The antioxidative effects of APHD on H_2_O_2_-induced PC12 cells. The chemical structure of APHD (**A**). Cell viabilities at different APHD concentrations in PC12 cells (**B**) and in H_2_O_2_-induced PC12 cells (**C**). Pretreatment with APHD inhibited LDH release (**D**) and decreased MDA concentrations (**E**). APHD increased the concentrations of GSH (**F**), GSH-Px (**G**), and SOD (**H**). APHD reduced the production of intracellular ROS (**I** and **J**). Data have the expressions of mean ± SD, *n* = 3. Distinct letters (a, b, c) represent significant differences at *p* < 0.05 employ the Compact Letter Display (CLD) methodology

The chemical structure of APHD is shown in Fig. 1A. A model of H_2_O_2_-induced PC12 cells was established to investigate the antioxidative properties of APHD. APHD displayed no cytotoxicity at concentrations up to 20 µM in both PC12 cells and SH-SY5Y cells (Fig. 1B and Fig. S1). Pretreatment with various concentrations of APHD reversed the cellular injury caused by H_2_O_2_ (Fig. 1C), and the effects of 10 µM and 20 µM treatments were comparable to that of the positive control TBHQ. Intracellular ROS levels were measured by flow cytometry. APHD reduced the release of LDH and the concentration of MDA while increasing the concentrations of GSH, GSH-Px, and SOD (Fig. 1D–H). As shown in Fig. 1I and J, APHD at 10 µM and 20 µM significantly reduced the production of intracellular ROS. Compared with 10 µM treatment, the 20 µM treatment showed an excellent inhibitory effect on ROS. However, APHD at 5 µM did not showed the protective effect. In sum, APHD relieves the oxidative stress injury in a dose-dependent manner, and 20 µM treatment showed the outstanding antioxidant effect in H_2_O_2_-induced PC12 cells.

### APHD reverses the oxidative stress injury in OGD/R-induced PC12 cells

**Fig. 2:**
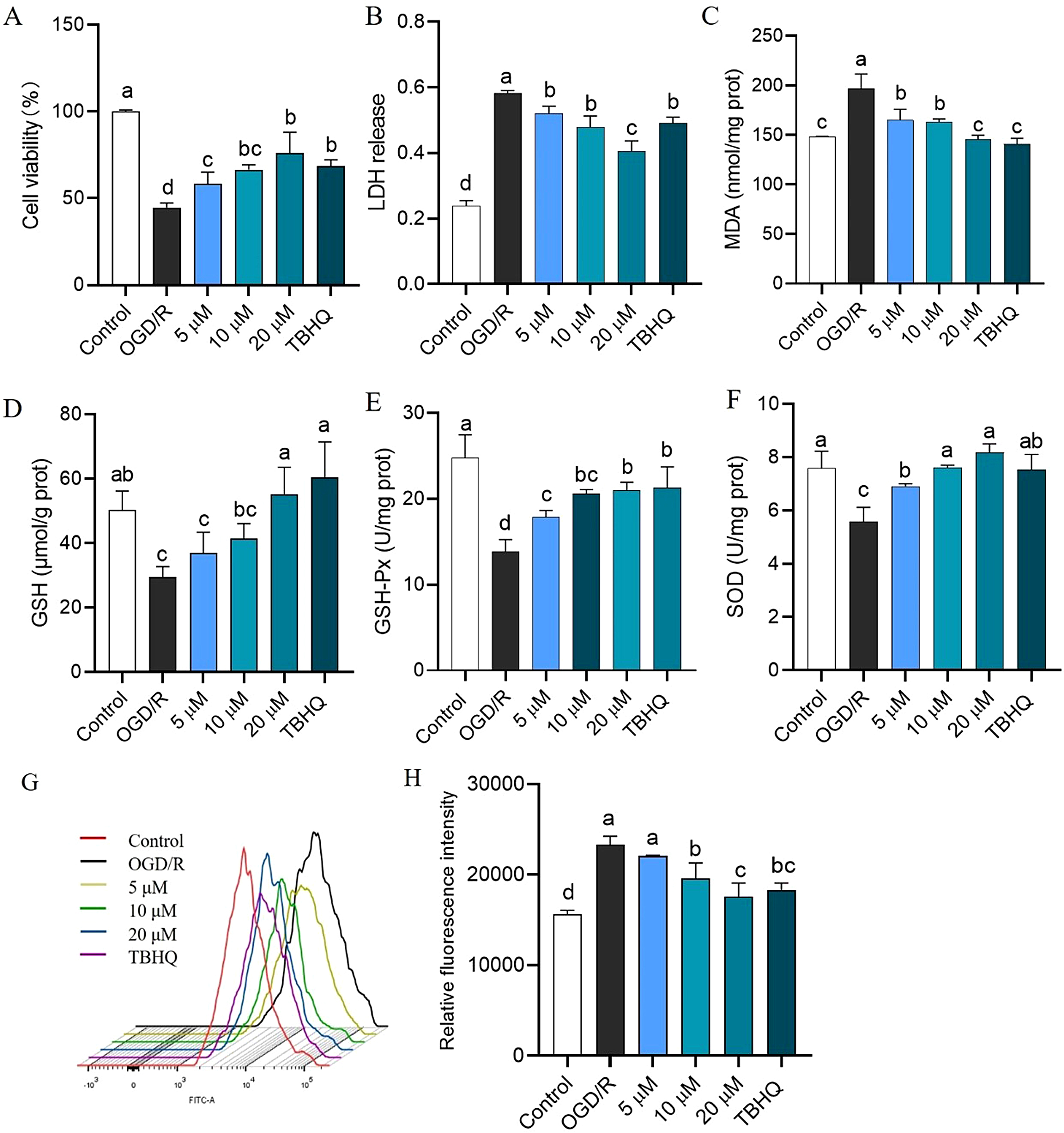
Protective effects of APHD on the damage to PC12 cells induced by OGD/R. The cell viabilities of different concentrations with APHD in PC12 cells subjected to OGD/R condition (**A**). Pretreated with APHD inhibited the LDH release (**B**) and decreased the concentration of MDA (**C**). APHD increased the concentrations of GSH (**D**), GSH-Px (**E**) and SOD (**F**). APHD down-regulated the production of intracellular ROS (**G**-**H**). Data have the expressions of the mean ± SD, *n* = 3. Distinct letters (a, b, c) represent significant differences at *p* < 0.05 employ the Compact Letter Display (CLD) methodology

Then, OGD/R induced PC12 cells were utilized to mimic cerebral ischemia injury in vitro. Cell viability decreased following OGD/R treatment. Pretreatment with 5 µM, 10 µM and 20 µM APHD, as well as TBHQ, mitigated this reduction (Fig. 2A). APHD downregulated the release of LDH and the concentration of MDA, while upregulating the concentrations of GSH, GSH-Px, and SOD (Fig. 2B–F). Flow cytometry results showed that APHD also decreased the production of intracellular ROS in a dose-dependent manner, which consistent with the results of APHD in H_2_O_2_-induced PC12 cells (Fig. 2G–H), indicating that APHD inhibited oxidation linked to cerebral ischemia injury. Our results showed that APHD of 20 µM exhibited a prominent neuroprotective effect against cerebral ischemia injury even better than the positive agent TBHQ. APHD have potential as candidates for novel neuroprotective agents.

### APHD shows the potential to activate Keap1-Nrf2/HO-1 signaling cascade

**Fig. 3:**
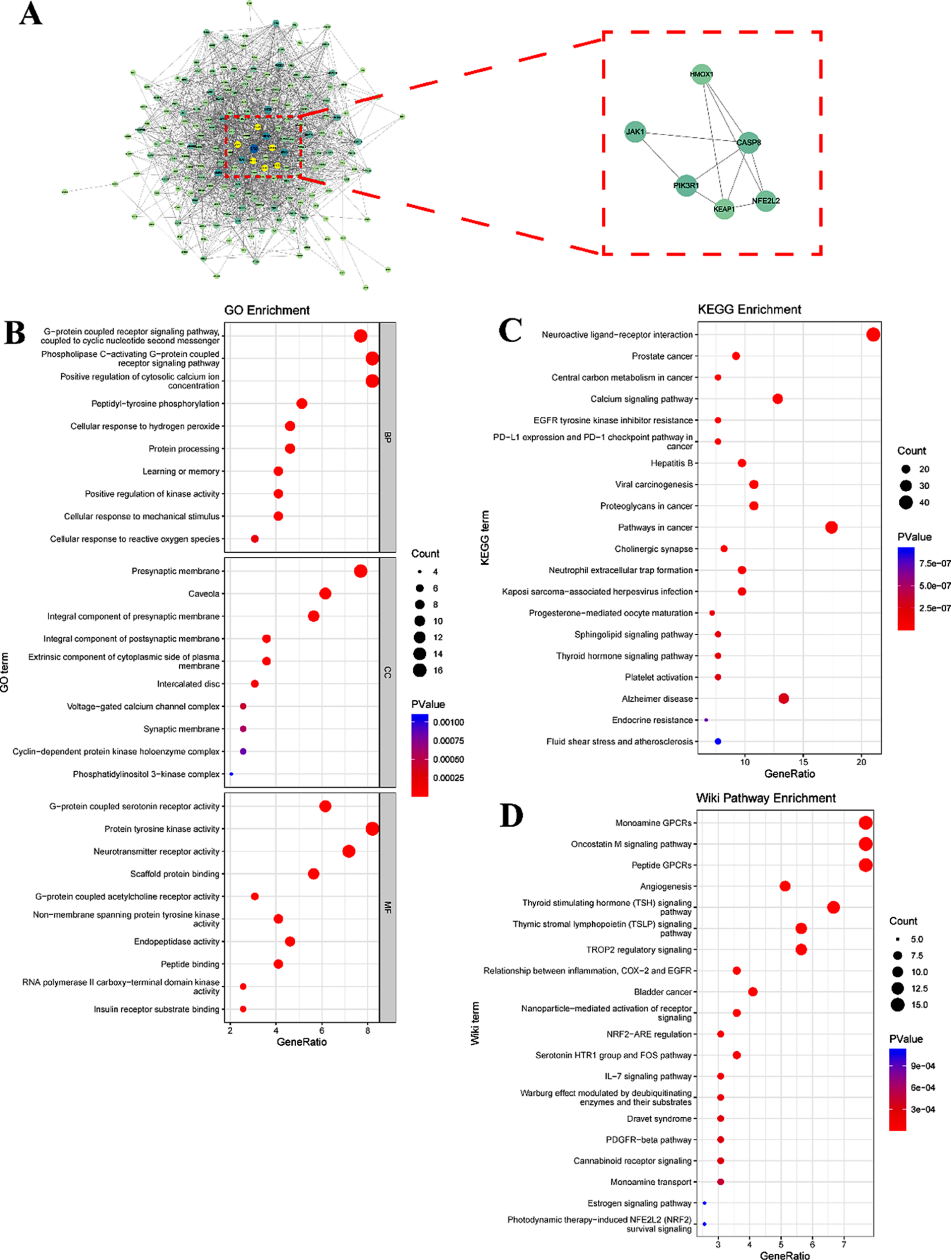
Potential targets and enriched pharmacologic terms associated with APHD. (**A**) The PPI network and hub genes of APHD-related targets. (**B**) The top 10 terms in biological process (BP), cellular component (CC), and molecular function (MF) for GO enrichment. (**C**) The top 20 terms in KEGG enrichment. (**D**) The top 20 terms in WikiPathways enrichment

After target mining and preprocessing, 193 proteins were identified as APHD-regulated targets. The PPI network for APHD-regulated targets comprised 193 nodes and 1,343 edges (Fig. 3A), indicating 1,343 interactions among these proteins. Six hub targets were identified based on network topology analysis, including Keap1 (shown as the gene name of KEAP1), Nrf2 (shown as the gene name of NFE2L2), HO-1 (shown as the gene name of HMOX1), caspase8 (shown as the gene name of CASP8), PI3K (shown as the gene name of PIK3R1) and JAK1. GO enrichment analysis was performed on APHD-regulated targets to probe gene functions, while KEGG and WikiPathways enrichment analyses were conducted to investigate the primary signaling cascades. Terms related to oxidative stress and the nervous system, such as ‘cellular response to reactive oxygen species,’ ‘presynaptic membrane,’ and ‘neurotransmitter receptor activity,’ were noted in the BP, CC, and MF in GO enrichment (Fig. 3B). Likewise, diseases and signaling cascades related to oxidative stress, including neuroactive ligand-receptor interaction and the Nrf2 signaling cascade, were represented in KEGG and WikiPathways enrichment analyses (Fig. 3C and D). In summary, APHD exhibits potential antioxidant capabilities that may be mediated by the Nrf2 signaling cascade.

**Fig. 4:**
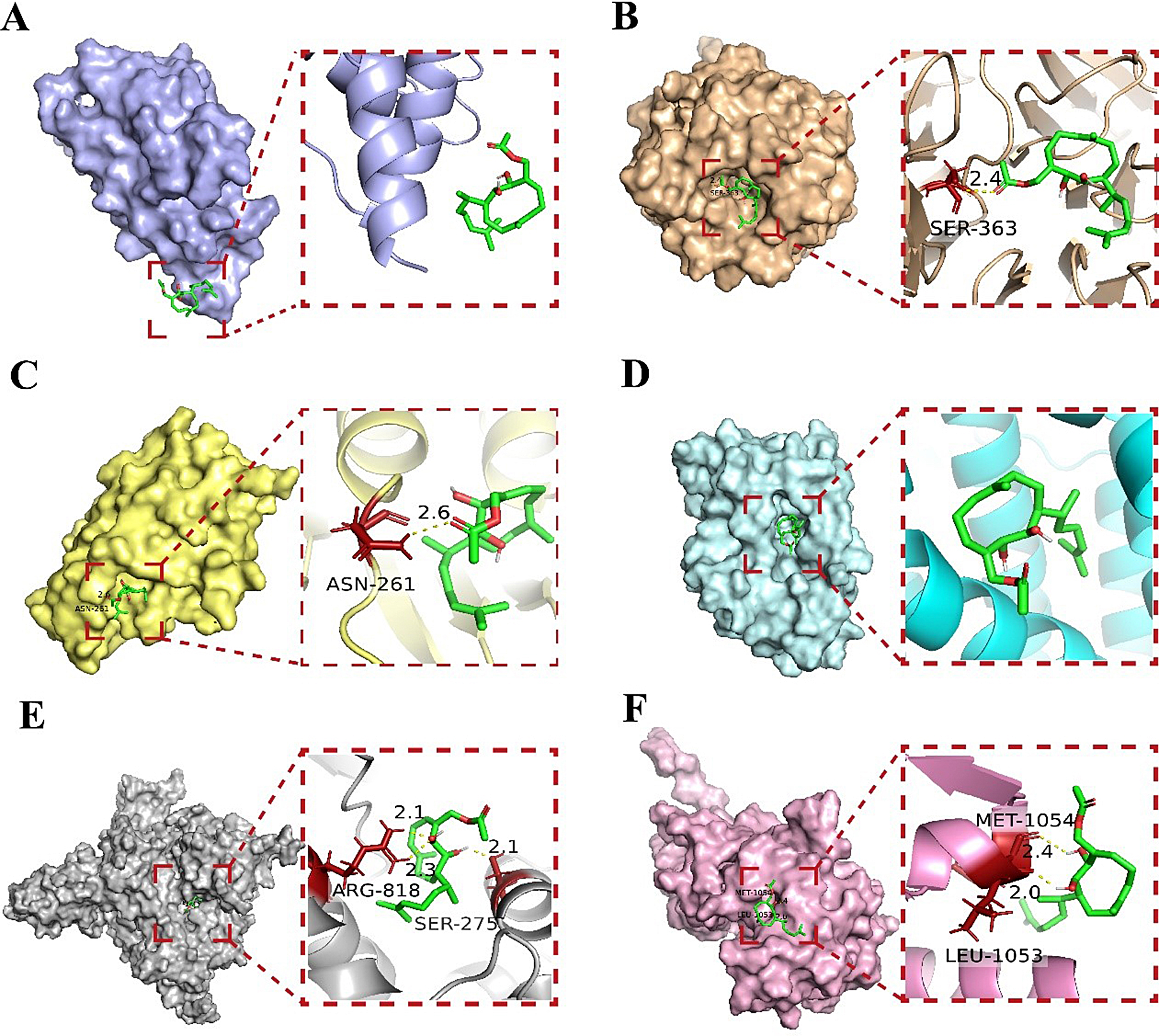
The potential binding conceptions of APHD-receptors. The APHD docking modes with keap1 BTB domain (-1.8 kcal/mol, **A**), keap1 kelch domain (-7.8 kcal/mol, **B**), CASP8 (-5.5 kcal/mol, **C**), HMOX1 (-6.2 kcal/mol, **D**), PIK3R1 (-6.5 kcal/mol, **E**), and JAK1 (-4.3 kcal/mol, **F**)

To identify the direct target modulated by APHD, molecular docking was utilized to calculate the binding affinity between each of the six hub proteins and the ligand (Fig. 4). Docking results indicated that APHD could bind to the BTB domain of Keap1 (PDB ID: 7exi), the Kelch domain of Keap1 (PDB ID: 4in4), caspase 8 (PDB ID: 6px9), HO-1 (PDB ID: 1xjz), PI3K (PDB ID: 5xgi), and JAK1 (PDB ID: 4ehz) with binding energies of -1.8, -7.8, -5.5, -6.2, -6.5, and − 4.3 kcal/mol, respectively. The lowest binding energy was observed with the APHD-Keap1 Kelch domain interaction, suggesting that the carbonyl oxygen of APHD forms a polar bond with the Serine (SER) 363 residue through a hydrogen bond in the Kelch pocket. This suggests that APHD may disrupt the Keap1-Nrf2 binding, inhibit the negative regulation of Nrf2, and thereby increase the concentration of free Nrf2.

### APHD regulates the Keap1-Nrf2/HO-1 cascade in H_2_O_2_-induced PC12 cells

**Fig. 5:**
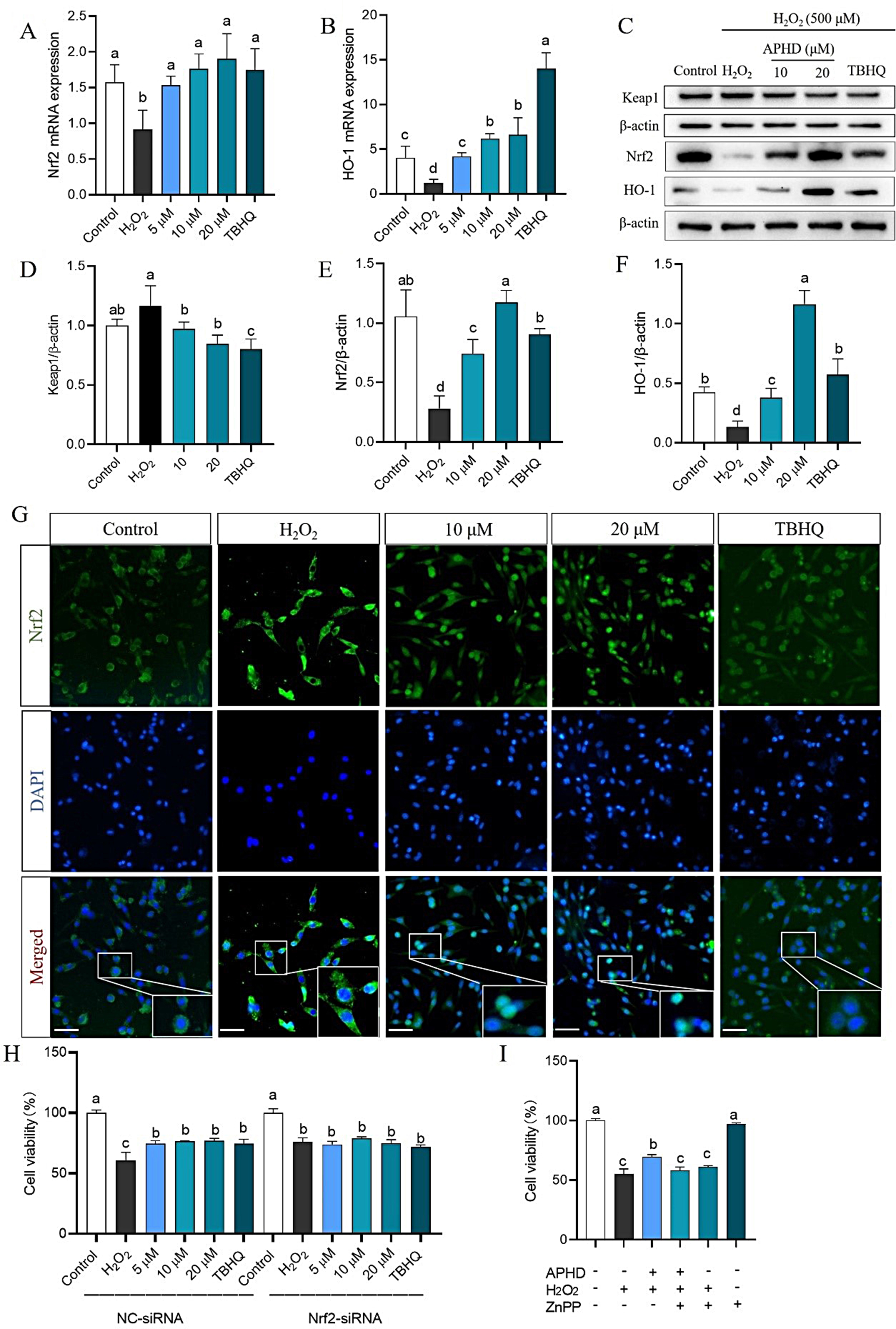
APHD activates the Keap1/ Nrf2/ HO-1 signaling cascade. APHD enhanced the mRNA concentrations of Nrf2 (**A**) and HO-1 (**B**), as well as the protein concentrations of Keap1, Nrf2, and HO-1 (**C**-**F**). APHD promotes Nrf2 nuclear translocation (**G**). The movement of Nrf2 into the cell nucleus was observed using an immunofluorescence test. Nrf2 was identified with a targeted antibody that emits green fluorescence. Cell nuclei were stained with DAPI, which appears blue. Representative images of the combined fluorescence and magnified views are presented. The scale marker indicates 50 micrometers, and the merge has been enlarged 400 times. Changes of cell viability after interference with Nrf2 siRNA (**H**). Treated with Znpp (a HO-1 inhibitor) reversed the antioxidative effect of APHD (I). Data have the expressions of the mean ± SD, *n* = 3. Distinct letters (a, b, c, d) represent significant differences at *p* < 0.05 employ the Compact Letter Display (CLD) methodology

Based on network pharmacology and molecular docking results, the impact of APHD on the Keap1-Nrf2/HO-1 cascade was confirmed. PCR results demonstrated that APHD at different concentrations enhanced the mRNA expression of Nrf2 and HO-1 (Fig. 5A and B). This was accompanied by increases in Nrf2 and HO-1 protein concentrations and a reduction in Keap1 protein concentration by Western-blot assay (Fig. 5C–F). Furthermore, immunofluorescence assays revealed that APHD could promote Nrf2 nuclear translocation (Fig. 5G). The nuclear translocation of Nrf2 triggers the activation of downstream antioxidant factors, producing an antioxidant effect. Subsequently, to determine whether the protective effect of APHD is dependent on Nrf2, we silenced the Nrf2 gene. Cell survival was measured via MTT assay to assess the compound’s protective effect. The cytoprotective effect of all concentrations in APHD was abrogated when Nrf2 was silenced, indicating that APHD’s antioxidant effect is mediated through Nrf2 (Fig. 5H). To further ascertain whether HO-1 contributed to the antioxidant effect of APHD, the HO-1 inhibitor ZnPP was used. Administration of APHD and ZnPP together had no impact on cellular survival relative to the H_2_O_2_ group (Fig. 5I), indicating that the antioxidative action of APHD could be attributed to the stimulation of the Keap1-Nrf2/HO-1 sequence.

### APHD activates the Keap1-Nrf2/HO-1 cascade in OGD/R-induced PC12 cells

**Fig. 6:**
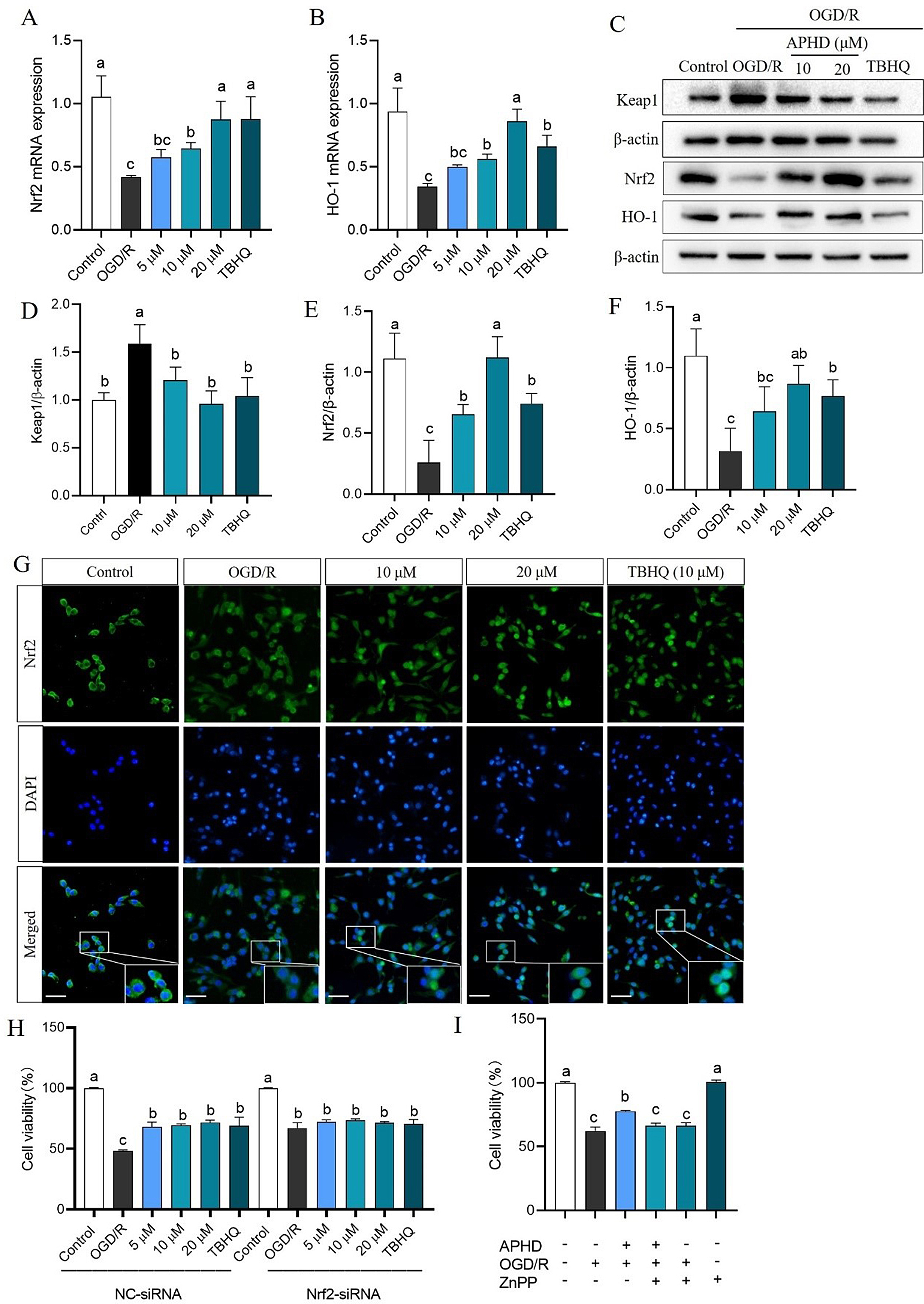
APHD activates the Keap1/Nrf2/HO-1 signaling cascade in damage to PC12 cells induced by OGD/R. APHD increased the mRNA concentrations of Nrf2 (**A**) and HO-1 (**B**), as well as the protein concentrations of Keap1, Nrf2, and HO-1 (**C**–**F**). APHD promotes Nrf2 nuclear translocation (**G**), as detected by immunofluorescence assay. Nrf2 was labeled with specific antibody (green) and nuclei with DAPI (blue). Representative images of merged and enlarged views are displayed. Scale bar = 50 μm, and the magnification of merge is 400×. Treatment with ZnPP, an HO-1 inhibitor, reversed the antioxidant effect of APHD (**H**). Cell viability changes after Nrf2 siRNA interference (**I**). Data have the expressions of mean ± SD, *n* = 3. Distinct letters (a, b, c) represent significant differences at *p* < 0.05 employ the Compact Letter Display (CLD) methodology

We have confirmed that APHD exerts a certain protective effect on OGD/R-induced PC12 cells, however, the mechanism of APHD against cerebral ischemia remains unclear. Consequently, the effect of APHD on the Keap1-Nrf2/HO-1 cascade was also confirmed in PC12 cells subjected to OGD/R condition. APHD upregulated the mRNA and protein concentrations of Nrf2 and HO-1, while downregulating the protein concentrations of Keap1. It is still the case that 20 μm APHD showed the best therapeutic effect, with a better effect than the positive agent TBHQ (Fig. 6A–F). Immunofluorescence assays additionally showed that APHD promoted Nrf2 nuclear translocation, and exerting an antioxidant effect (Fig. 6G). Nrf2 and HO-1 activation induced by APHD was nullified by Nrf2 silencing and by ZnPP treatment, respectively (Fig. 6H and I). These findings indicate that APHD may mitigate cerebral ischemia injury by activating the Keap1-Nrf2/HO-1 cascade.

## Discussion

Oxidative stress damage plays a role in numerous neurological diseases, including Alzheimer’s disease [26], Parkinson’s disease [27], and stroke [28]. In the management of ischemic stroke, the primary objective is to rapidly restore cerebral blood flow. However, reperfusion can further exacerbate damage to ischemic brain tissue, leading to a worse prognosis [29]. Oxidative stress is critically significant in the pathophysiology of cerebral ischemia. Consequently, the discovery of antioxidative agents represents a promising strategy for preventing cerebral ischemia. In our previous reports, several compounds isolated from the genus *Dictyota* have demonstrated excellent neuroprotective effects. APHD, a germacrane-type diterpene, was initially isolated from *Dictyota* with high abundance. To date, there have been no reports on the neuroprotective effects of APHD. Here, we investigated the neuroprotective effect of APHD in H_2_O_2_-induced PC12 cells and in PC12 cells subjected to OGD/R. This study has, for the first time, shown that APHD can significantly inhibit oxidative stress via the Keap1-Nrf2/HO-1 pathway and is expected to become a potential therapeutic against cerebral ischemia.

In recent years, natural products have made significant advances in managing ischemic stroke [30], with many exhibiting anti-inflammatory and antioxidative properties in cerebral ischemia models [31, 32]. Hydrogen peroxide, a powerful oxidant, can trigger an intracellular oxidative stress response, resulting in cellular damage. To model this oxidative stress in the brain, we initially established H_2_O_2_-induced PC12 cells. Following the pioneering work of Goldberg et al. [33, 34] in creating an ischemic model of primary nerve cells using a box-type hypoxia chamber, the OGD/R model has been widely used to study cerebral ischemia-reperfusion injury and serves as an important in vitro method for investigating cerebral ischemic damage. We exposed PC12 cells to OGD/R to simulate the conditions of cerebral ischemic injury. APHD improved cell viability and reduced the release of LDH and MDA, as well as increased the levels of antioxidant enzymes such as HO-1, GSH, GSH-Px, and SOD in both cell models. Furthermore, APHD decreased the level of intracellular ROS, thereby exerting a neuroprotective effect in this study. Additionally, we evaluated the effects of different concentrations of APHD (5, 10, and 20 µM) and found that its protective effect was dose-dependent. Overall, we determined that 20 µM of APHD provided the most effective protection, surpassing that of the positive agent TBHQ. These findings suggest that APHD could be considered a potentially effective neuroprotective agent.

**Fig. 7:**
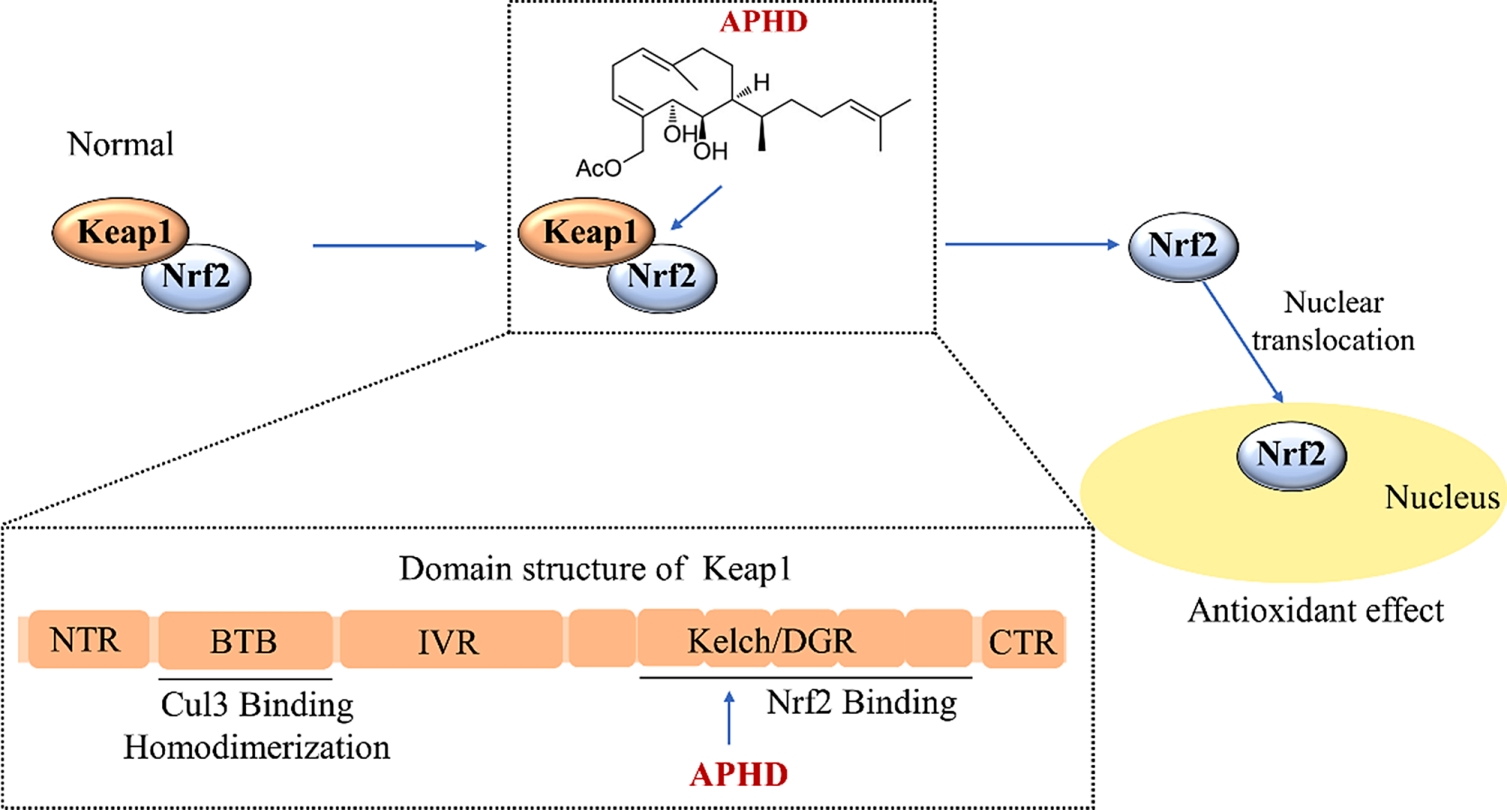
The mechanisms of Nrf2 activation by APHD. APHD interferes with the interplay between Keap1 (Kelch-like ECH-associated protein 1) and Nrf2 (NFE2-related factor 2) through binding to Kelch domain’s active pocket. Keap1 contains five domains: NTR (amino terminal region), BTB (broadcomplex/tramtrack/bric-a-brac), IVR (intervening region), Kelch (kelch domain of Nrf2) / DGR (double glycine repeat) and CTR (C-terminal region). APHD bound to the SER363 residue in the Kelch domain’s active pocket to exert the antioxidant effect

Oxidative stress significantly affects many disease processes, and involved in many pathways. To explore the potential mechanism, we have established network pharmacology to investigate the possible effects and pharmacological mechanisms of APHD. The findings suggest that APHD may have potential anti-cerebral ischemic activity, which could be associated with the Nrf2 cascade. There are different modes of Nrf2 activation for small molecules. The first type of Nrf2 agonist acts directly on Keap1, a negative regulator of Nrf2, by covalently binding to the active cysteine residue on Keap1 and inhibiting the interaction between Keap1 and Cul3. The Keap1 protein loses its ability to mediate Nrf2 protein’s ubiquitination and subsequent breakdown through the Cul3 protein. This effect increases the stable presence of Nrf2 protein and enhances the Nrf2 signaling cascade’s transcriptional activity [35, 36]. The second type of Nrf2 agonist interferes with the interplay between Keap1 and Nrf2, thereby freeing Nrf2 protein from Keap1’s negative regulatory effect and increasing the stable Nrf2 protein content [37, 38]. To investigate how APHD activates Nrf2, we employed molecular docking. APHD successfully bound to the SER363 residue in the Kelch domain’s active pocket but failed to bind to Cys151, suggesting that APHD may act in a manner similar to the second mode, interfering with the interplay between Nrf2 and Keap1 (Fig. 7). The design of activators targeting the Keap1-Nrf2 protein–protein interaction is becoming an increasingly important field of antioxidant discovery [39], and APHD is also expected to be a potent Nrf2 activator with neuroprotective effects. Therefore, our subsequent studies will use molecular interaction methods to further investigate the binding mode of APHD to Keap1 protein.

Based on the results of network pharmacology and molecular docking, we further explore whether APHD activates the Nrf2 pathway in both H_2_O_2_-induced PC12 cells and OGD/R-induced PC12 cells. In this study, qRT-PCR, western blot, and immunofluorescence were utilized to explore the Keap1-Nrf2/HO-1 signaling cascade in our research. APHD increased the mRNA levels and concentrations of Nrf2 and HO-1, as well as decreased the concentration of Keap1, while also promoting Nrf2 nuclear translocation. The neuroprotective effect of APHD was negated by Nrf2 silencing. Additionally, the administration of ZnPP, an HO-1 inhibitor, reversed the antioxidant effect of APHD. These results revealed that APHD is capable of activating the Keap1-Nrf2/HO-1 signaling cascade. The results were consistent with those of network pharmacology and molecular docking. Activating the Nrf2 signaling cascade, helping regulate the body’s redox balance, is a therapeutic strategy for various neurological diseases [40, 41]. Previous research demonstrated that oxidative stress is implicated in most neurological diseases and that the Keap1-Nrf2 cascade has the potential to defend against oxidative damage [42, 43]. Nrf2 can be activated through both Keap1-dependent and independent pathways, translocating to the nucleus and binding to its response elements to activate downstream antioxidant enzymes such as HO-1, GSH, GSH-Px, and SOD [44, 45]. This activation enhances the scavenging efficiency of ROS and OH·, mitigating oxidative stress damage. Our results suggested that APHD might act as a Nrf2 agonist and exert antioxidant effects against cerebral ischemia. Additionally, due to the limited quantity of APHD currently available, in vivo efficacy studies remain to be conducted. In summary, APHD regulated the concentrations of antioxidant enzymes, inhibited the release of LDH and MDA, and scavenged excessive ROS by activating the Keap1-Nrf2/HO-1 signaling cascade in PC12 cells subjected to H_2_O_2_ and OGD/R conditions.

## Conclusion

APHD, a germacrane-type diterpene primarily extracted from the brown algae belonging to the *Dictyota* genus, has shown a significant neuroprotective effect against oxidative damage in two cell models. The fundamental process through which APHD delivers its antioxidative effects involves Keap1-Nrf2/HO-1 signaling cascade activation. APHD is anticipated to be a potent antioxidant for addressing brain ischemic damage. This study provides evidence that marine algae are a valuable source of neuroprotectants and can serve as another example of the medicinal use of marine algae. This research offers a new perspective and scientific justification for the further exploration and utilization of *Dictyota* algae resource.

### Electronic supplementary material

Below is the link to the electronic supplementary material.

Supplementary Material 1

Supplementary Material 2

## Data Availability

No datasets were generated or analysed during the current study.
