# Supplementary material for: Neuroprotective effect of acetoxypachydiol against oxidative stress through activation of the Keap1-Nrf2/HO-1 pathway
Source: BMC Complement Med Ther. 2024 Apr 25;24:175. doi: 10.1186/s12906-024-04474-6 (PMC11044414; doi:10.1186/s12906-024-04474-6)
Supplement: Supplementary file 1 — Supplementary Material 1 [file 12906_2024_4474_MOESM1_ESM.docx]

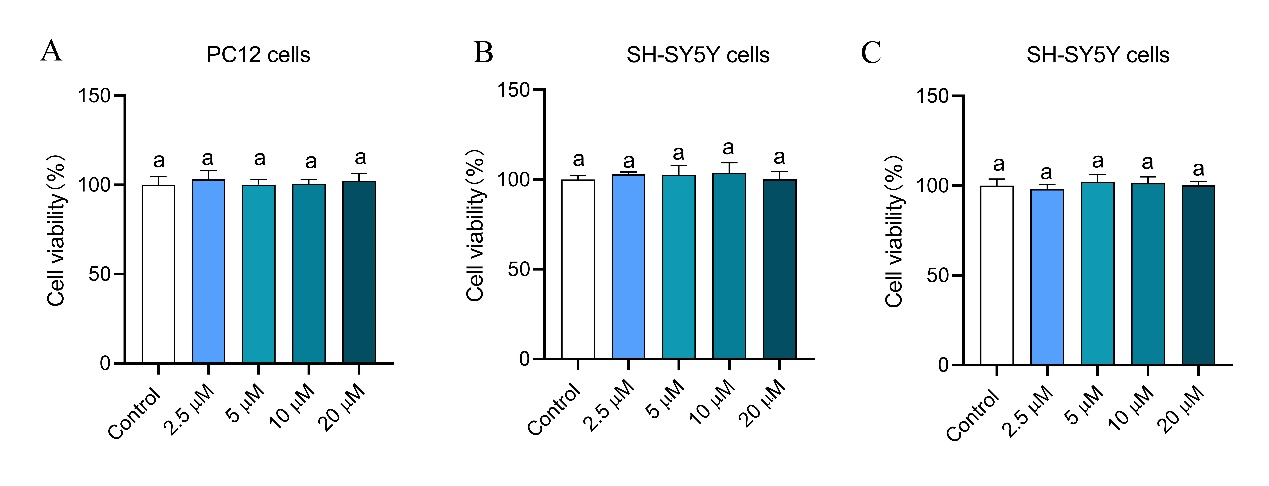


Fig. S1 Cytotoxicity test of APHD in two cells. Cell viabilities of APHD at different concentrations in PC12 cells by CCK8 assay (A). Cell viabilities of APHD at different concentrations in PC12 cells by MTT assay (B) and CCK8 assay (C). Statistically significant differences of the means across all conditions are indicated with compact letter display (CLD).
