# Supplementary figures and images for: Neuroprotective effect of acetoxypachydiol against oxidative stress through activation of the Keap1-Nrf2/HO-1 pathway
Source: BMC Complement Med Ther. 2024 Apr 25;24:175. doi: 10.1186/s12906-024-04474-6 (PMC11044414; doi:10.1186/s12906-024-04474-6)

Supplementary material for Western Blotting

**Fig. 5**

β-actin


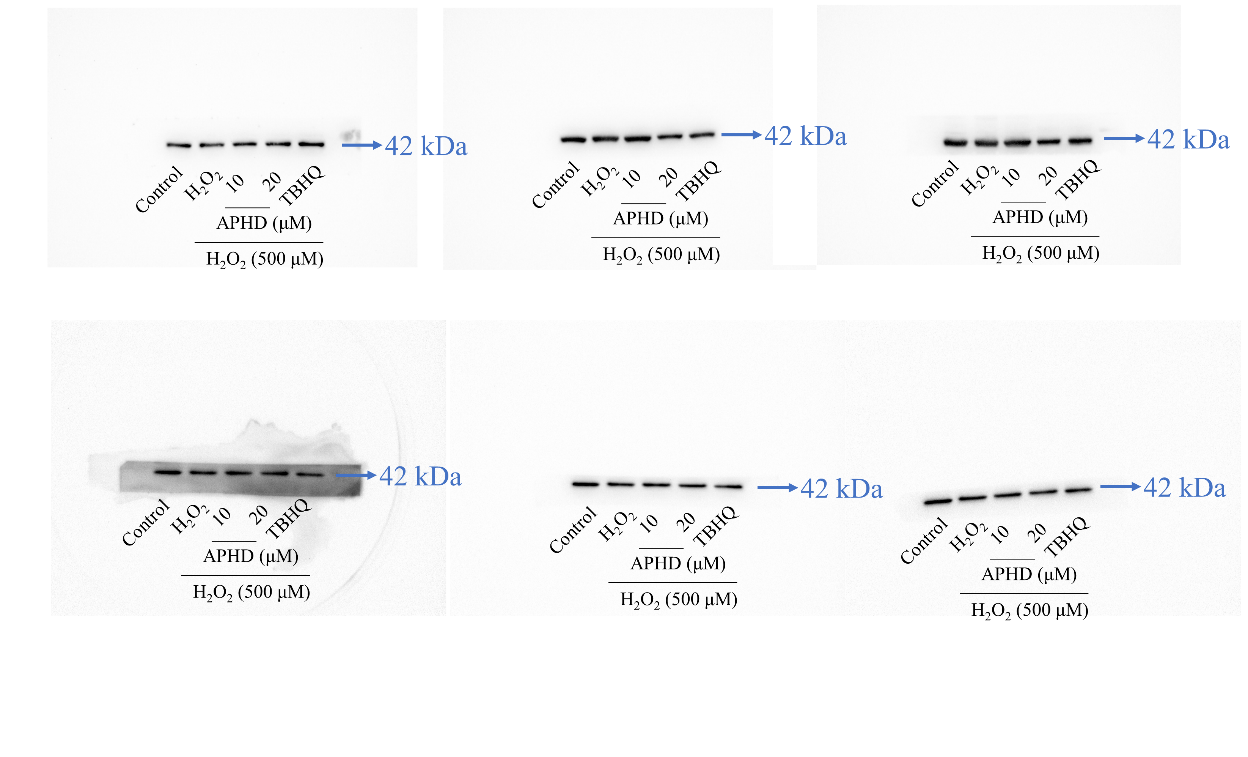


HO-1


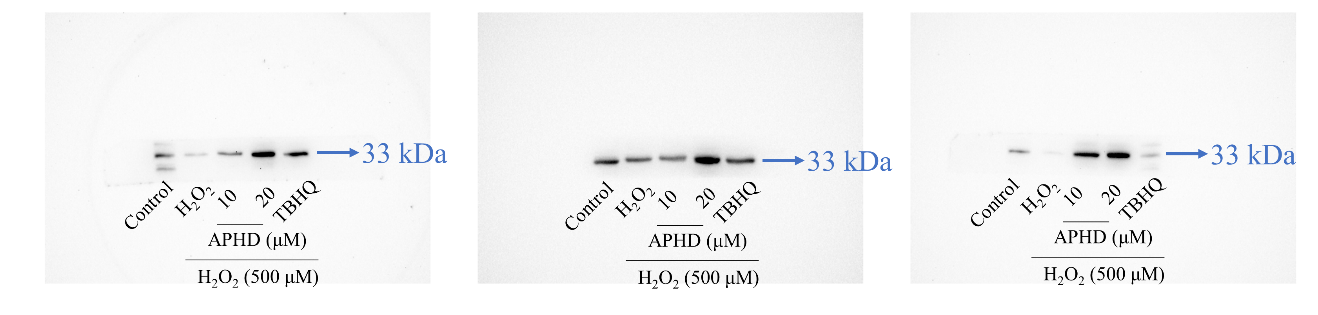


Nrf2


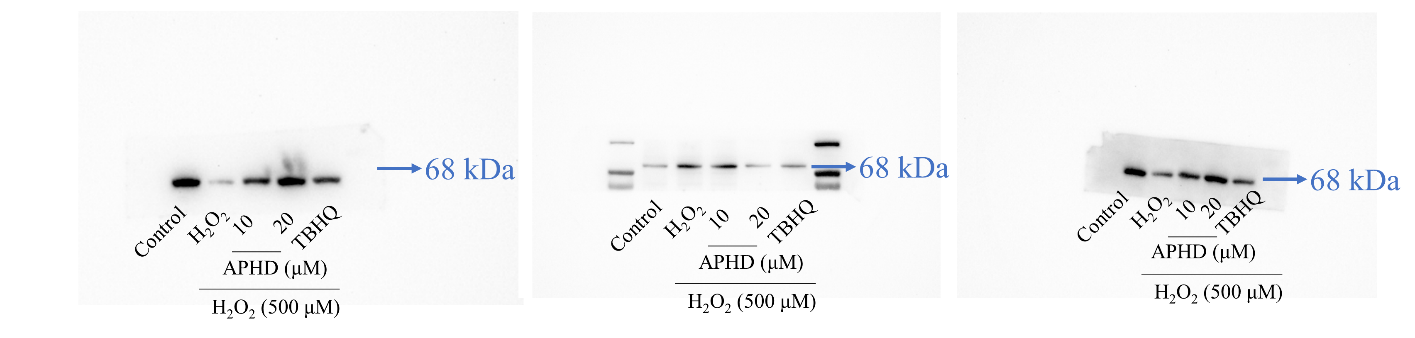


Keap1


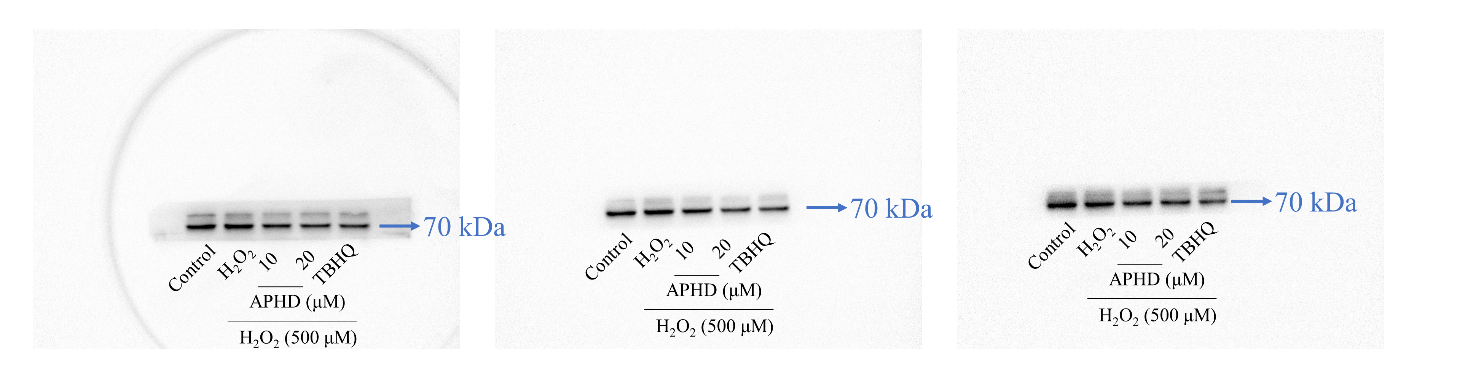


**Fig. 6**

β-actin


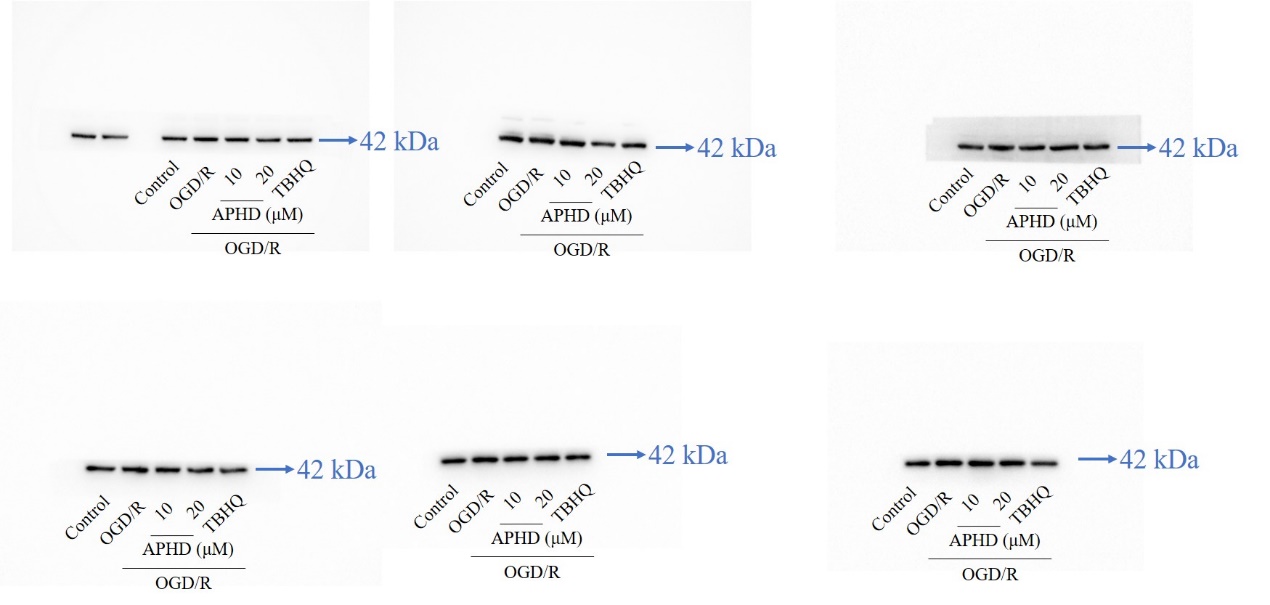


HO-1


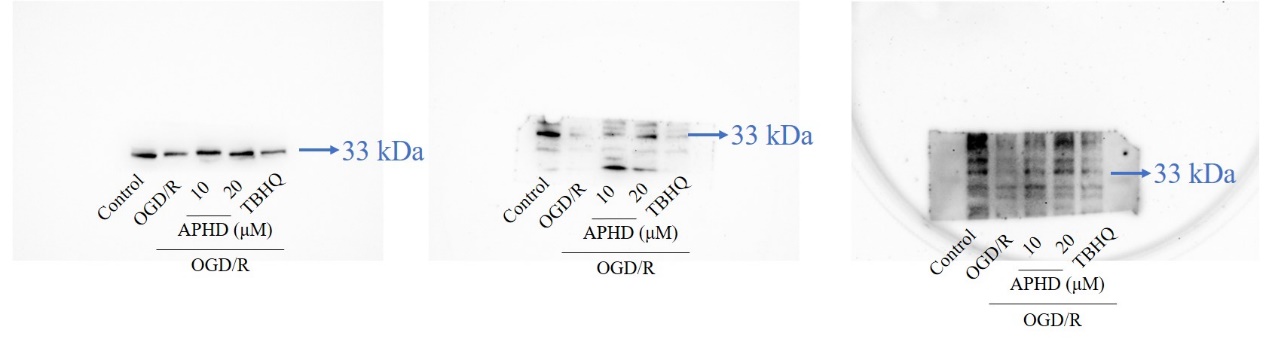


Nrf2


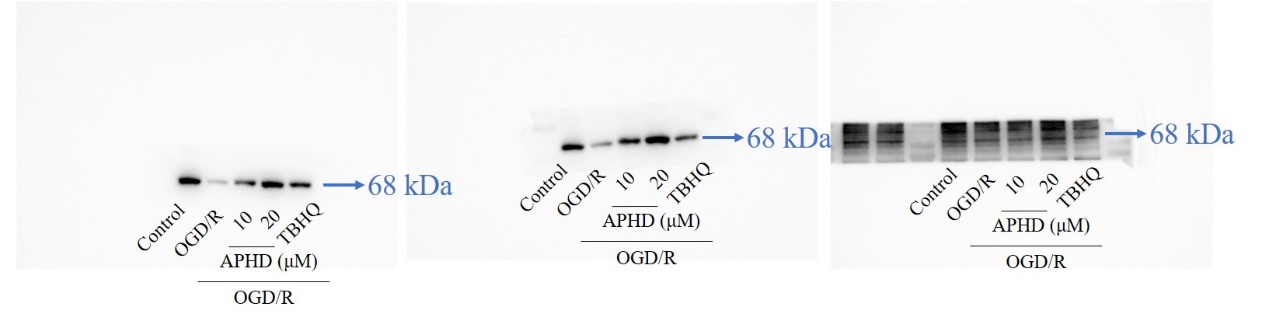


Keap1


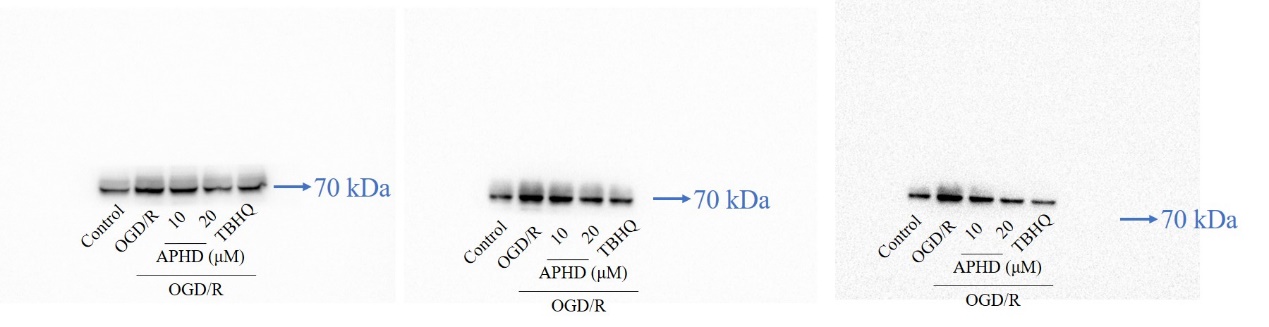

Supplement: Supplementary file 2 — Supplementary Material 2 [file 12906_2024_4474_MOESM2_ESM.docx]
